# Explant of the Aortic Endograft: Today’s Solutions, Tomorrow’s Problems

**DOI:** 10.14797/mdcvj.1176

**Published:** 2023-03-07

**Authors:** Alan B. Lumsden

**Affiliations:** 1 Methodist DeBakey Heart & Vascular Center, Houston Methodist, Houston, Texas, US

**Keywords:** graft explantation, abdominal aortic endograft, aneurysm enlargement, type 2 endoleaks

## Abstract

Type 2 endoleaks remain the Achilles heel of abdominal aortic endografting. They drive imaging costs and repeat intervention. We believe that after two endovascular interventions, patients should be considered for either graft explantation or graft salvage through an open abdominal exploration. Graft explantation has been associated with increased morbidity and mortality but remains necessary in the face of non-correctible type 1a endoleaks, graft failure, or graft infection. In the majority of cases AAA expansion due to persistent type 2 endoleak is the culprit. In this situation, open repair, with oversewing of the lumbar or inferior mesenteric arteries, can be accomplished providing the seal zones and component overall zones are adequate. This approach does not require aortic clamping. We provide detailed descriptions and videos to facilitate the surgeon in performing these complex procedures.

## Introduction

Recent years have seen a dramatic increase in the number of aneurysms treated with abdominal aortic endografts. Indeed, up to 70% of primary repairs are now performed with an abdominal aortic endograft. At the present time, a significant number of open aortic procedures reflect remedial secondary interventions. This represents two broad groups of patients: first, those in whom aneurysm enlargement continues to occur most often due to type 2 endoleaks; and second, those who have nonremedial type 1 endoleaks or graft infections (Video 1). These open procedures can be particularly challenging, especially in the latter group. Such operations are associated with higher morbidity and mortality.^1,2^ This article will address some of the technical challenges and management of these patients but will focus on practical challenges associated with removing these devices. For this reason, I include links to the extensive video library of DeBakey Cardiovascular Education. The videos herein can be found on the DeBakey Cardiovascular Education YouTube channel at https://www.youtube.com/c/HoustonMethodistDeBakeyCVEducation.

**Video 1:** Non-remedial type 1 endoleaks or graft infections. https://www.youtube.com/watch?v=IjCNBZyjVb8&t=3186s.

## Exposure and Salvage of Endograft

The indication for exposing and salvaging an endograft is an enlarging aneurysm from a type 2 endoleak. Of the two types of open aortic procedures requiring remedial secondary interventions, this type is a bit more frequent and less technically challenging.^3,4,5^ Typically, these patients present many years after placement of an endograft and have had frequent and numerous interventions for aneurysm growth directed at occluding either the lumbar arteries or the inferior mesenteric artery. Table 1 describes the types of approaches. Generally, when a plethora of approaches have been tried, it usually implies that none of them work particularly well. This reflects the complex nature of type 2 endoleaks.

**Table 1:** Techniques for embolizing type 2 endoleaks.


Transarterial: superior mesenteric to inferior mesenteric ascending lumbar to the lumber arteries

Perigraft

Transgraft

Transcaval

Translumbar

Transabdominal


When evaluating the patient who has failed catheter therapy for type 2 endoleaks, we carefully evaluate the proximal and distal seal zones to ensure that there is a good seal. Typically, this is a minimum of 10 mm proximally and ideally 20 mm distally. A good seal requires a minimum length, over which there is complete wall opposition between the endograft and the wall of the aorta or iliac artery. It is especially important to ensure there is no proximal type 1 leak. Confidence in the integrity of the seal zones is fundamental to the approach described here. Increasingly, we have been using dynamic computed tomography (CT) scans to evaluate this. Additionally, the surgeon must evaluate overlap zones within modular devices. Spending time preoperatively with a good CT angiogram (CTA), allowing the surgeon to map out where the offending lumbars enter the back of the aneurysm, proves very beneficial once the aneurysm is opened. Lumbar bleeding, which occurs at the proximal end of the aneurysm immediately below the neck, can be particularly difficult to expose due to the presence of the endograft. Having a plan for where to look for these lumbar arteries saves time and minimizes blood loss. Once these factors have been evaluated, a decision for open repair with graft salvage is based on patient comorbidities and abdominal hostility.

Our hospital’s most common approach is a long midline incision with standard exposure of the abdominal aorta. We expose the neck of the aneurysm in order to place a clamp in the infrarenal position. If this is necessary, we typically use padded Fogarty clamps. As we have gained more and more experience with graft salvage, we minimize the dissection of the iliac arteries if we are satisfied with the seal zone. Video 2 (at 2 minutes, 17 seconds) shows an aneurysm being opened. Emergency endoluminal control inside the endograft can be obtained if necessary. It is notable that the aortic wall after endograft placement is often thickened and inflamed, although the reason for this is not understood.

**Video 2:** (at 2 minutes, 17 seconds) shows an aneurysm being opened; see also at https://www.youtube.com/watch?v=w-2iuAFrFH4.

Once adequate proximal control has been obtained, we make a vertical incision through the wall of the aneurysm, which is then extended circumferentially immediately below the distal extent of the proximal seal zone. This entire process is performed using electrocautery due to the aforementioned thickening and inflammation in the aneurysmal wall. As an adjunct, we frequently use a running locking stitch to provide hemostasis in the aneurysmal wall. After the aneurysm is opened, the operator is presented with organized thrombus. Typically, there is no active bleeding at this point; however, if the inferior mesenteric artery is one of the sources, then oversewing the orifice from inside the aneurysm may be required early.

We use an empty sponge stick and finger to gently remove the organized thrombus, taking care not to grasp the endograft graft in this process. One needs to work around in the graft, which can lay anywhere within the sac. Most commonly, it lies in the anterior sac, but the operator must determine this by review of the preoperative CT scan. This is important because one must get behind the graft to address the lumbar arteries. As thrombus is removed, bright red blood is encountered when entering the endoleak cavity. Cell saver suction is recommended since bleeding can be fairly dramatic. Entering the cavity, which also can be located on the CT scan, allows one to follow the bleeding down to a source; when there are many, this can be confusing.

We attempt to remove all the thrombus down to the wall of the aneurysm. The ligation of all the lumbar artery origins is performed with 2-0 silk pop-off sutures. Once again, knowing how many lumbar one is looking for, and their location, is very helpful. The most difficult lumbars to address are those that occur from the aneurysm wall immediately after the neck flares into the aneurysm because the operator is constrained by the presence of the endograft. It certainly crosses the operator’s mind that there is a type 1 endoleak, which is why a diligent review of the preoperative CT scan is reassuring. Once the immediate bleeding is controlled, it becomes necessary to spend time removing all the thrombus from the aortic wall to prevent delayed hemorrhage.

Since the intervention is being performed for an enlarging aneurysm, there is usually more than adequate sack to cover the graft. If bleeding from the aortic wall is ongoing, this is the point when we run and lock a suture along the length of the wall. Prior to closure, the operator also may decide to trim some of the aortic wall to provide a tighter closure over the underlying device.

We used to fill the aneurysm sack with omentum, but I do not believe this is always necessary. Another adjunct we routinely use, which can be performed early after exposure or after evacuation of the hematoma, is to place three or four horizontal mattress pledgeted 3-0 prolene sutures through the wall of the aorta, encompassing the underlying device. These are placed at least in the 12 o’clock, 3 o’clock, and 9 o’clock positions. Our thought process is to ensure secure anchoring of the proximal aortic device. Depending on the adequacy of the iliac arteries, we may place similar pledgeted sutures distally. Closure of the abdomen then continues in standard fashion.

## Explantation of Abdominal Aortic Endografts

The absolute indication for explanting an abdominal aortic endograft is graft infection. This is associated with an aortoduodenal fistula in up to 25% of cases.^6^ Other indications include persistent type 1 endoleak and failure of the integrity of the device. However, increasingly in the case of proximal leak or device failure, endovascular salvage techniques are available. This discussion primarily addresses aortic graft infection.^7,8^

Once the diagnosis of graft infection has been established, attention focuses on the technique for graft removal (Table 2). This decision centers on (1) the type of graft, particularly if there is suprarenal fixation; (2) whether there is an aortoenteric fistula; (3) whether an aortoiliac or aortofemoral reconstruction is necessary; and (4) the type of reconstruction to be performed (Table 5).

**Table 2:** Strategies for dealing with late complications of endografts.


No graft explant	Type 2 endoleaks

Partial graft explant	Sew to proximal or iliac remnants retention of suprarenal bare metal stent

Total graft explantation	


Careful evaluation of the proximal seal zone, perirenal segment, and visceral segment of the aorta is fundamental to performing this procedure adequately and safely. These decisions must be made against the background of patient comorbidities and risk. In some cases, a full thoracoabdominal approach may be ideal to permit complete device explantation. However, many patients cannot tolerate such a procedure. In general, we find that leaving behind the metallic suprarenal struts is a reasonable compromise to permit this procedure to be performed through a transabdominal approach alone. A midline laparotomy should be adequate if the plan is to remove all material below the renal arteries, although a retroperitoneal approach provides a little more latitude in moving clamps up into the visceral and supraceliac segment. Video 3 (at 4 minutes, 12 seconds) shows the technique for graft removal. If there are any doubts, we recommend obtaining supraceliac control prior to entering the aorta. Quality of the aorta, where the plan is to sew a graft, is important in being able to hold sutures and perform this operation with some degree of safety. Unfortunately, this is not always easy to determine before the procedure. The presence of an aortoduodenal fistula is managed in essentially the same way as a fistula complicating open repair, which is not addressed in this manuscript.

**Video 3:** (at 4 minutes, 12 seconds) shows the technique for graft removal; see also at https://www.youtube.com/watch?v=jNxe1hP6FF4.

**Video 4:** (at 7 minutes, 9 seconds) shows the technique for obtaining supraceliac control; see also at https://www.youtube.com/watch?v=D8oOjt1X_Ns.

The following describes steps for graft explantation:

1. Strongly consider placing ureteric stents.
2. Obtain supraceliac control. Video 4 (at 7 minutes, 9 seconds) shows this technique.
3. Expose the aneurysm and the iliac arteries essentially in the same way as the open aortic aneurysm repair, although this is likely to be more challenging due to periaortic inflammation.
4. In the overwhelming majority of cases, divide the left renal vein.
5. Get circumferential control of each renal artery and prepare for suprarenal clamping.
6. Perform clamping proximally and distally using padded Fogarty clamps, which mold better around the aorta and the underlying endograft.
7. Open the aneurysm in a relatively standard fashion with longitudinal electrocautery, and extend circumferentially immediately distal to the seal zone.
8. As the aneurysm is entered, be prepared to encounter purulence, infected thrombus, and occasionally bleeding from the lumbar artery, which need to be suture ligated.
9. Take cultures from any purulence, thrombus, and the graft before sending for culture.
10. Usually, divide the endograft using heavy scissors. This is done by choosing an area between the stent rings, if possible.
11. Rotate the proximal end of the device toward the abdomen and gradually work along the stent graft to free it from the proximal attachment zone.
12. Free up the graft in the perirenal space. A number of techniques can be used to do this. Video 5 (at 6 minutes, 16 seconds) depicts one technique for using a transected syringe to advance into the aorta and attempt to remove any suprarenal barbs. Fairly frequently, this also involves cutting the suprarenal struts with wire cutters.^9^
13. We have no personal experience in dealing with infected devices that have deployed endoanchors, which would markedly increase the complexity of completely removing all fabric.
14. Focus on ensuring that adequate aortic wall is left after the explantation to permit safe suturing of a proximal aortic anastomosis.
15. Reconstructive options (Table 5) include the use of cryopreserved aortic graft (Video 6, at 1 minute and 49 seconds) and autogenous femoral vein graft (Video 7, at 5 minutes and 31 seconds), although we almost universally use rifampin-soaked Dacron (Table 3). (See also https://www.youtube.com/watch?v=SZmjBSDZhTo.)
16. Create the proximal anastomosis by ensuring that adequate bites of healthy aortic tissue are available, which is key for this step.
17. The distal anastomosis may be equally challenging although, in general, the explantation of the entire endograft is feasible. The surgeon must decide whether the tissue is healthy enough for an iliac anastomosis or whether the bypass should be extended to the common femoral arteries. Again, careful examination of the preop CT scan will guide the distal end of the stent graft relative to the bifurcation.
18. Since this dissection around the iliacs also may be challenging due to the perivascular inflammation, the presence of ureteric stents can be very helpful. They can be palpated, but not easily.
19. Almost universally, whenever feasible, mobilize the omentum to cover the graft and close the aneurysm sac. Video 8 (at 5 minutes, 7 seconds) shows how to create an omental flap.
20. Because these complex procedures rarely follow a straightforward postoperative course, I recommend treating these patients with lifelong antibiotics tailored to the intraoperative cultures.

**Video 5:** (6 minutes, 16 seconds) depicts a technique for using a transected syringe to advance into the aorta and attempt to remove any super real barbs; see also at https://www.youtube.com/watch?v=ql409_cBJ7c&t=33s.

**Video 6:** (at 1 minute and 49 seconds) shows cryopreserved aortic graft; see also at https://www.youtube.com/watch?v=3Cpm7Bz_sRQ.

**Video 7:** (at 5 minutes and 31 seconds) shows autogenous femoral vein graft; see also at https://www.youtube.com/watch?v=tPyQqO0Q9iU.

**Video 8:** (at 5 minutes, 7 seconds) shows how to create an omental flap; see also at https://www.youtube.com/watch?v=GnG0WoOLvHY&t=31s.

**Table 3:** Rifampin protocol.


600 mg rifampin in 500 mL saline

Ensure completely dissolved

Soak graft for 45 minutes


The postoperative management of these patients may be very complicated, and one needs to carefully follow the renal function. Main concerns long-term are for recurrent infection of any retained endograft and reinfection of the graft used for reconstruction. Retaining portions of the graft is associated with high risk of infection.^1,10^

Numerous alternate techniques may be of value in certain circumstances (Table 4).^11^ For example, if sutures will not hold in the perirenal space, it may be necessary to move the anastomosis up closer to the superior mesenteric artery. In this situation, a hepatorenal and splenorenal bypass may be of value but will markedly increase the magnitude of the operation, so it must be determined in advance.^12^ If an anastomosis cannot be performed, then one alternative, although somewhat discredited, is to oversew the aortic stump (Video 9, at 3 minutes, 45 seconds), and perform extra anatomical bypass (Table 5). This, of course, has delayed risk of stump blowout, especially when the aorta is diseased. Direct puncture of the graft with placement of a proximal balloon control is another alternative for challenging proximal dissection.^13^

**Video 9:** (at 3 minutes, 45 seconds) demonstrates oversew of the aortic stump; see also at https://www.youtube.com/watch?v=Z6WKuPFYnbQ.

**Table 4:** Adjunctive techniques.


Hepatorenal/splenorenal

Abdominal debranching

Ascending aorta to visceral bypass

Oversew stump - axillobifemoral bypass

Endograft puncture and proximal balloon control


**Table 5:** Reconstructive options.


In situ reconstruction	Rifampin soaked Dacron Aorta iliac Aorta femoral Autogenous femoral vein (NAIS) Cryopreserved aortoiliac

Extraanatomical	Axillo femoral bypass, oversew aortic stump


The procedure, when performed for type 2 endoleak with graft salvage, can be performed safely. However, procedures performed for infected endografts, in which explantation is required, are associated with up to a 20% mortality rate.^14,15^

## Beyond the Abdominal Aorta

Techniques for graft explantation have been developed for almost every part of the aorta,^16,17^ although it is beyond the scope of this article to address all of these variances. As endografts begin to be used for the thoracoabdominal segment and the arch, salvage operations will continue to evolve and become more complex. Table 6 lists additional videos of procedures we have performed for graft explantation in the thoracic aorta (for example, Video 10 at 1 minute, 54 seconds). At the present time, the overwhelming number of explants continue to be performed in the abdomen. However, no device has ever been implanted that is free of complications demanding explantation, which remains a challenging problem with high morbidity and mortality. Indeed, today’s solutions certainly generate new problems for tomorrow.

**Video 10:** (at 1 minute, 54 seconds) shows graft explantation in the thoracic aorta; see also at https://www.youtube.com/watch?v=uDtK3Be6P84.

**Table 6:** Aortic videos. EVAR: endovascular aneurysm repair; TEVAR: thoracic endovascular aortic repair


PROCEDURE	VIDEO LINK

The infected aortic endograft: diagnosis and therapy	https://www.youtube.com/watch?v=IjCNBZyjVb8&t=3186s

Open repair type 2 endoleak after EVAR	https://www.youtube.com/watch?v=w-2iuAFrFH4

EVAR explantation	https://www.youtube.com/watch?v=EwUN72QBNnE

Explantation of infected aortic endograft with suprarenal fixation	https://www.youtube.com/watch?v=ql409_cBJ7c&t=33s

Removal of infected stent graft, aortobiiliac rifampin soaked graft	https://www.youtube.com/watch?v=SZmjBSDZhTo

Neoaortoiliac reconstruction	https://www.youtube.com/watch?v=tPyQqO0Q9iU

Oversewing an aortic stump	https://www.youtube.com/watch?v=Z6WKuPFYnbQ

Supra celiac exposure	https://www.youtube.com/watch?v=D8oOjt1X_Ns

Omental flap	https://www.youtube.com/watch?v=GnG0WoOLvHY&t=31s

Open aortobifemoral bypass - retroperitoneal approach	https://www.youtube.com/watch?v=jNxe1hP6FF4

Left visceral rotation	https://www.youtube.com/watch?v=KVuMiAuw5zc

Femoral vein harvest	https://www.youtube.com/watch?v=3Cpm7Bz_sRQ

Pseudo coarctation of aorta - removal of collapsed thoracic endograft	https://www.youtube.com/watch?v=Kr4GS80-4b0

TEVAR explantation and aortoesophageal fistula repair	https://www.youtube.com/watch?v=uDtK3Be6P84


## Key Points

- Type 2 endoleak is a major cause of late AAA expansion.
- Many endografts can be salvaged at the time of exploration.
- The list provided on the fourth page will help determine whether graft salvage should be considered.
- Careful localization of the lumbar arteries facilitates exploration and reduces blood loss.
